# A Theoretical Investigation of the Polyaddition of an AB_2_+A_2_+B_4_ Monomer Mixture

**DOI:** 10.3390/polym16030426

**Published:** 2024-02-03

**Authors:** Sergei V. Karpov, Artem Iakunkov, Dmitry A. Chernyaev, Vladimir G. Kurbatov, Georgiy V. Malkov, Elmira R. Badamshina

**Affiliations:** 1 Department of Polymers and Composites, Federal Research Center of Problems of Chemical Physics and Medicinal Chemistry of Russian Academy of Sciences, 1 Academician Semenov Avenue, Chernogolovka 142432, Russia; chernyayevda@icp.ac.ru (D.A.C.); kurbatovvg@list.ru (V.G.K.); gmalkov@icp.ac.ru (G.V.M.); badamsh@icp.ac.ru (E.R.B.); 2 School of Engineering Sciences in Chemistry, Biotechnology and Health, KTH Royal Institute of Technology, SE-100 44 Stockholm, Sweden; iakunkov@kth.se

**Keywords:** hyperbranched polymers, degree of branching, co-polyaddition, gel point, AB_2_+A_2_+B_4_ monomer mixture

## Abstract

Hyperbranched polymers (HBPs) are widely applied nowadays as functional materials for biomedicine needs, nonlinear optics, organic semiconductors, etc. One of the effective and promising ways to synthesize HBPs is a polyaddition of AB_2_+A_2_+B_4_ monomers that is generated in the A_2_+CB_2_, AA′+B_3_, A_2_+B′B_2_, and A_2_+C_2_+B_3_ systems or using other approaches. It is clear that all the foundational features of HBPs that are manufactured by a polyaddition reaction are defined by the component composition of the monomer mixture. For this reason, we have designed a structural kinetic model of AB_2_+A_2_+B_4_ monomer mixture polyaddition which makes it possible to predict the impact of the monomer mixture’s composition on the molecular weight characteristics of hyperbranched polymers (number average (DP_n_) and weight average (DP_w_) degree of polymerization), as well as the degree of branching (DB) and gel point (p_g_). The suggested model also considers the possibility of a positive or negative substitution effect during polyaddition. The change in the macromolecule parameters of HBPs formed by polyaddition of AB_2_+A_2_+B_4_ monomers is described as an infinite system of kinetic equations. The solution for the equation system was found using the method of generating functions. The impact of both the component’s composition and the substitution effect during the polyaddition of AB_2_+A_2_+B_4_ monomers on structural and molecular weight HBP characteristics was investigated. The suggested model is fairly versatile; it makes it possible to describe every possible case of polyaddition with various monomer combinations, such as A_2_+AB_2_, AB_2_+B_4_, AB_2_, or A_2_+B_4_. The influence of each monomer type on the main characteristics of hyperbranched polymers that are obtained by the polyaddition of AB_2_+A_2_+B_4_ monomers has been investigated. Based on the results obtained, an empirical formula was proposed to estimate the p_g_ = p_A_ during the polyaddition of an AB_2_+A_2_+B_4_ monomer mixture: p_g_ = p_A_ = (−0.53([B]_0_/[A]_0_)^1/2^ + 0.78)υAB_2_ + (1/3)^1/2^([B]_0_/[A]_0_)^1/2^, where (1/3)^1/2^([B]_0_/[A]_0_)^1/2^ is the Flory equation for the A_2_+B_4_ polyaddition, [A]_0_ and [B]_0_ are the A and B group concentration from A_2_ and B_4_, respectively, and υAB_2_ is the mole fraction of the AB_2_ monomer in the mixture. The equation obtained allows us to accurately predict the p_g_ value, with an AB_2_ monomer content of up to 80%.

## 1. Introduction

The synthesis and investigation of properties of hyperbranched polymers (HBPs) represents one of the most rapidly advancing areas in polymer science. They have a wide range of applications due to the number of unique features compared to the linear and cross-linked polymers, including high solubility, thermodynamic compatibility, low viscosity, high sorption capacity, and a high content of functional groups [1,2,3]. HBPs are widely applied nowadays as functional materials for biomedicine needs [4,5], nonlinear optics [6,7], organic semiconductors [8,9], and flame-retardant materials [10,11], among others.

**Scheme 1:**
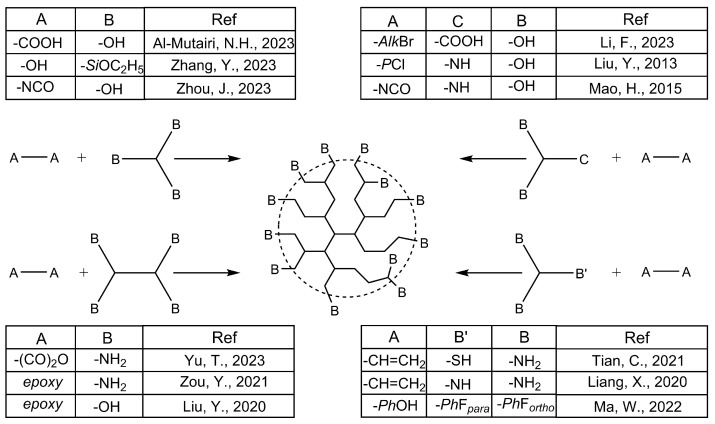
Synthesis of HBP by polyaddition of the monomer types A_2_+B_3_ [23,24,25], A_2_+B_4_ [26,27,28], A_2_+CB_2_ [29,30,31], and A_2_+B′B_2_ [32,33,34].

One of the key ways to obtain HBPs is homo-polyaddition of AB_m_-type monomers [12,13,14]. The primary advantage of polyaddition of AB_m_-type monomers is that it does not lead to gelation [15], allowing for a production of high-molecular-weight (MW) polymers with a degree of branching (DB) of 0.5 [16]. However, obtaining AB_m_-type monomers often involves a complex organic synthesis; moreover, there are some considerable complications arising in the process of isolation and purification of these monomers containing highly reactive groups [17,18]. This poses a notable barrier to the practical application of HBPs that are obtained through the aforementioned methods. For this reason, co-polyaddition of monomer mixtures of different types, for example, A_2_+B_3_, A_2_+B_4_, etc., have found wider application [19,20,21,22,23,24,25,26,27,28] (Scheme 1).

The introduction of this method has enabled a significant expansion of the range of monomers that are under use and also the carrying out of polyaddition as a single-step reaction. It is a known fact that this kind of co-polyaddition eventually results in the formation of a three-dimensional structure at a specific juncture, commonly referred to as the critical gelation conversion, or gel point (p_g_). To determine the p_g_ value in these Flory systems, Equation (1) was offered [35].

(1) $$\alpha =\frac{rp_{A}^{2}\rho }{1-rp_{A}^{2}\left(1-\rho \right)}=\frac{p_{B}^{2}\rho }{r-p_{B}^{2}\left(1-\rho \right)}$$

where r = [A]_0_/[B]_0_, ρ is the ratio of B (or A) groups in branched units to the total number of these groups, and p_A_ and p_B_ are the conversions of A and B groups, respectively.

In general, p_g_ = max (p_A_, p_B_). Hereinafter, when [A]_0_/[B]_0_ > 1, p_g_ = p_B_, because p_A_ < p_B_ in that range. Correspondingly, if [A]_0_/[B]_0_ < 1, then p_g_ = p_A_, and when [A]_0_/[B]_0_ = 1, p_A_ = p_B_ = p_g_.

To reduce the p_g_ value, co-polyaddition of asymmetric monomers (A_2_+CB_2_, A_2_+B′B_2_, AA′+B_3_, A_2_+C_2_+B_3_) was introduced [29,30,31,32,33,34,35,36,37,38,39] (Scheme 1). These approaches made it possible to shift the gel point, since more AB_2_ monomers were formed, and therefore, it was possible to obtain polymers with an increased MW. To describe the polyaddition of A_2_+CB_2_ monomers, a number of simulations have been developed [40,41] to predict polydispersity index (PDI) values depending on the ratio of reactants (Equation (2)).

(2) $$\mathrm{PDI}=\frac{{\mathrm{DP}}_{w}}{{\mathrm{DP}}_{n}}=\frac{(1+1/\lambda -2p_{A}[2p_{B}p_{C}+p_{B}^{2}+\lambda +p_{C}^{2}/(2\lambda )+p_{B}^{2}/\lambda +1+4p_{B}+2p_{C}]}{(1+1/\lambda {)}^{2}(\lambda -p_{B}^{2}-2p_{B}p_{C})}$$

where DP_w_ and DP_n_ are the weight average and the number average degree of polymerization, p_A_, p_B_, p_C_ are conversions of A, B, and C groups, and λ is the initial ratio of A_2_ and CB_2_ monomer concentrations.

**Scheme 2:**
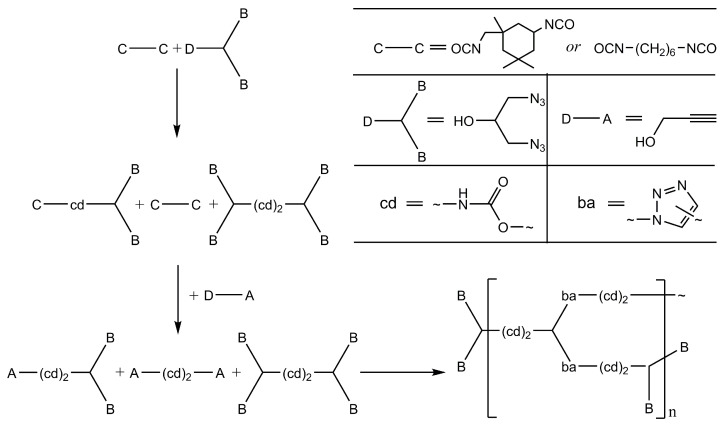
Synthesis of HBP by polyaddition of the monomers of AB_2_+A_2_+B_4_ type [36,37], where ba is the product of interaction between A and B groups, cd is the product of interaction between C and D groups.

Previously, we successfully implemented an approach to obtain HBPs, using polyaddition of the AB_2_+A_2_+B_4_ monomer mixture with controlled contents of each constituent [42,43] (Scheme 2). That technique can also be applied to the co-polyaddition of asymmetric monomers due to the formation of AB_2_ monomers.

Despite the fact that the AB_2_+A_2_+B_4_ monomer mixture can be obtained during the polyaddition of A_2_+CB_2_ monomers, there is a lack of current theories and ideas to adequately describe every possible combination of these monomers in the mixture. The methods described above prevent obtaining a complete picture of the impact of each constituent of the AB_2_+A_2_+B_4_ monomer mixture on HBP formation.

Moreover, positive or negative substitution effects taking place during polyaddition and described in a number of experimental papers [44,45,46,47] would significantly affect both the MW and the structural characteristics of the resulting polymers. The manifestation of a positive substitution effect, e.g., in the Friedel–Crafts aromatic substitution reaction of AB_2_, leads to the production of fully branched HBPs [38]. The manifestation of a negative substitution effect, e.g., during the production of hyperbranched polyesters by co-polycondensation of an AB_2_-type monomer and B_4_- and B_6_-type polyfunctional cores, leads to a decrease in the MW of the final product [39]. There is no doubt that the substitution effect will also affect the value of p_g_ in cases where it may be less than 1.

The kinetic Monte Carlo method and molecular dynamics simulations are widely used nowadays to investigate the evolution of the structure of hyperbranched polymers and polymer networks [48,49,50]. At the same time, the conventional kinetic method that has proven itself for the investigation of HBP formation currently remains of interest [51,52,53,54,55].

Given all the facts above, we aim to develop a new structural kinetic model of the polyaddition of an AB_2_+A_2_+B_4_ monomer mixture, taking into account the potential manifestation of the substitution effect during polyaddition. Additionally, it would enable us to determine the impact of each system constituent on the structural and molecular weight parameters of HBPs.

## 2. Calculation Section

### 2.1. Design of the Kinetic–Structural Model

To describe the AB_2_+A_2_+B_4_ system, it is essential to establish certain assumptions and conditions. These will provide a framework for describing various reactions and types of resulting compounds that may emerge.

The assumptions are as follows:

- Flory assumption, i.e., function group reactivity is independent of the chain length;
- System homogeneity;
- No solvent impact.

**Figure 1:**
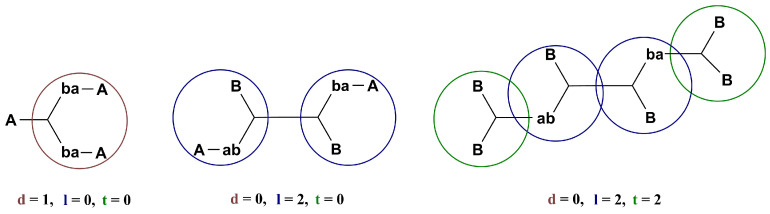
Structural units in the AB2+A2+B4 system, where ba/ab is the product of interaction between A and B groups.

The designed model is based on the concept of homo-polyaddition of AB_2_-type monomers [55]. To describe the AB_2_+A_2_+B_4_ system properly, it is also necessary to add a new parameter to the ones that were employed in [55] (the number of linear (*l*) and terminal (*t*) units). That is the number of dendritic units (*d*) (Figure 1).

The addition of the *d* unit results in the introduction of a new kind of compound, A_n_, which cannot be described accurately by *t* and *l* parameters only, since the number of A groups depends on *d:* A_max_ = *d* + 2.

The number of A groups in a macromolecule is equal to A_max_ when *l* units are formed without any *t* ones. In case of the formation of a *t* unit, the number of A groups in a macromolecule is 2 less, while the number of *d* units is only 1 less than in the compound A_n_ (Figure 1). So, the amount of A groups during the *t* unit formation equals −1 × *t* + A_max_, resulting in the following equation describing a real case of polyaddition as A = *d* + 2 − *t*.

**Scheme 3:**
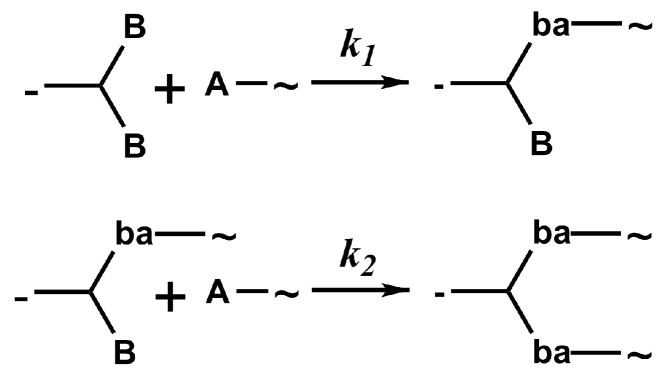
Positive and negative substitution effects during the polyaddition of the AB_2_+A_2_+B_4_ monomer mixture, where ba is the product of interaction between A and B groups.

The substitution effects, occurring when the polyaddition of AB_2_+A_2_+B_4_ monomer mixture takes place, are included in the structural kinetic model (Scheme 3).

The reactivity of B groups belongs to *t* units and can be determined by the *k*_1_ rate constant, whereas one of the B groups from *l* units is included in the *k*_2_ rate constant. B groups can be provided by either AB_2_ or B_4_ monomers and also by the interaction products of these monomers and with an A_2_-type monomer. Thus, in the case of *k*_1_/*k*_2_ < 1, a positive substitution effect takes place, whereas in the case of *k*_1_/*k*_2_ > 1, there is a negative substitution effect.

Alterations in all structural parameters during the studied reaction can be described as a set in Equation (3):

(3) $$\begin{array}{c} R\left(l,t,d\right)+R\left(l\prime ,t\prime ,d\prime \right)\overset{k_{1}}{\to }R\left(l+l\prime +1,t+t\prime -1,d+d\prime \right)\\ R\left(l,t,d\right)+R\left(l\prime ,t\prime ,d\prime \right)\overset{k_{2}}{\to }R\left(l+l\prime -1,t+t\prime ,d+d\prime +1\right)\\ A_{2}+R\left(l,t,d\right)\overset{k_{1}}{\to }R(l+1,t-1,d)\\ A_{2}+R\left(l,t,d\right)\overset{k_{2}}{\to }R\left(l-1,t,d+1\right), \end{array}$$

where R(*l*,*t*,*d*) is a concentration of macromolecules with *l*—linear, *t*—terminal, and *d*—dendritic units.

The introduction of additional reactions with the A_2_-type monomer is necessary to describe the initial conditions properly. According to the set of reactions (3), the endless kinetic equation can be defined by the following Equation (4), with initial conditions being [A_2_] = [A_2_]_0_, *R*(0,1,0) = [AB_2_]_0_, and *R*(0,2,0) = [B_4_]_0_, and the other *R*(*l*,*t*,*d*) = 0:

(4) $$\begin{array}{c} \frac{\mathrm{dR}(l,t,d)}{d\tau }=-(d+2-t)R(l,t,d)\left\{2k_{1}\sum_{l,t,d}^{\infty }\mathrm{tR}(l,t,d)+k_{2}\sum_{l,t,d}^{\infty }\mathrm{lR}(l,t,d)\right\}-(2k_{1}t+k_{2}l)R(l,t,d)\times \\ \times \left\{2[A_{2}]+\sum_{l,t,d}^{\infty }(d+2-t)R(l,t,d)\right\}+2k_{1}\left\{\sum_{l_{1}=0}^{l}\sum_{t_{1}=0}^{t}\sum_{d_{1}=0}^{d}(d+2-t)R(l_{1},t_{1},d_{1})\left(t-t_{1}+1\right)R(l-l_{1}-1,t-t_{1}+1,d-d_{1})\right.+\\ \left.+\sum_{l_{1}=0}^{l}\sum_{t_{1}=0}^{t}\sum_{d_{1}=0}^{d}(d+2-t)R(l_{1},t_{1},d_{1})\left(t-t_{1}+1\right)R(l-l_{1}-1,t-t_{1}+1,d-d_{1})\right\}+\\ +2k_{1}2[A_{2}]R(l-l_{1}-1,t-t_{1}+1,d-d_{1})+\\ +k_{2}\left\{\sum_{l_{1}=0}^{l}\sum_{t_{1}=0}^{t}\sum_{d_{1}=0}^{d}(d+2-t)R(l_{1},t_{1},d_{1})\left(l-l_{1}+1\right)R(l-l_{1}+1,t-t_{1},d-d_{1}-1)\right.+\\ +\left.\sum_{l_{1}=0}^{l}\sum_{t_{1}=0}^{t}\sum_{d_{1}=0}^{d}(d+2-t)R(l_{1},t_{1},d_{1})\left(l-l_{1}+1\right)R(l-l_{1}+1,t-t_{1},d-d_{1}-1)\right\}+k_{2}2[A_{2}]R(l-l_{1}+1,t-t_{1},d-d_{1}-1) \end{array}$$

The solution to the systems containing a large number of differential equations can only be achieved through the convolution of these equations. One of the simplest ways to accomplish this is by employing generating functions:

(5) $$\Phi \;\equiv \;\sum_{l,t,d}^{\infty }R(l,t,d)s^{l}p^{t}n^{d},$$

where *s*, *p*, and *n* are random variables.

Equation (4) can then be convolved with the *Φ* function into a shorter one (6):

(6) $$\frac{d\Phi }{d\tau }=-(n\Phi_{n}+2\Phi -p\Phi_{p})(2k_{1}T+k_{2}L)-(D+2N-T+2[A_{2}])(2k_{1}p\Phi_{p}+k_{2}s\Phi_{s})+\left(n\Phi_{n}+2\Phi -p\Phi_{p}+2[A_{2}]\right)(2k_{1}s\Phi_{p}+k_{2}n\Phi_{s})$$

Consequently, we can switch from Equation (6) to the moments of the generating function *Φ* (7):

(7) $$\begin{array}{c} \Phi (1,1,1)=\sum_{l,t,d}^{\infty }R(l,t,d)\equiv N,\;\Phi_{s}(1,1,1)=\sum_{l,t,d}^{\infty }\mathrm{lR}(l,t,d)\equiv L,\;\Phi_{p}(1,1,1)=\sum_{l,t,d}^{\infty }\mathrm{tR}(l,t,d)\equiv T,\\ \Phi_{n}(1,1,1)=\sum_{l,t,d}^{\infty }\mathrm{dR}(l,t,d)\equiv D,\;\Phi_{\mathrm{ss}}\equiv \frac{\partial^{2}\Phi }{\partial s^{2}}=\sum_{l,t,d}l^{2}R(l,t,d)-L,\\ \Phi_{\mathrm{sp}}\equiv \frac{\partial^{2}\Phi }{\partial s\partial p}=\sum_{l,t,d}\mathrm{ltR}(l,t,d),\;\Phi_{\mathrm{sn}}\equiv \frac{\partial^{2}\Phi }{\partial s\partial n}=\sum_{l,t,d}\mathrm{ldR}(l,t,d),\;\Phi_{\mathrm{pp}}\equiv \frac{\partial^{2}\Phi }{\partial p^{2}}=\sum_{l,t,d}t^{2}R(l,t,d)-T,\\ \Phi_{\mathrm{pn}}\equiv \frac{\partial^{2}\Phi }{\partial p\partial n}=\sum_{l,t,d}\mathrm{tdR}(l,t,d),\;\Phi_{\mathrm{nn}}\equiv \frac{\partial^{2}\Phi }{\partial n\partial n}=\sum_{l,t,d}d^{2}R(l,t,d)-D, \end{array}$$

and then the set of differential equations (8) for moments of the generating function *Φ* can be obtained from the Equations (6) and (7):

(8) $$\left\{\begin{array}{c} \frac{\mathrm{dN}}{d\tau }=-(D+2N-T)(2k_{1}T+k_{2}L)\\ \frac{\mathrm{dL}}{d\tau }=-(D+2N-T+2[A_{2}])(k_{2}L-2k_{1}T)\\ \frac{\mathrm{dT}}{d\tau }=-2k_{1}T(D+2N-T+2[A_{2}])\\ \frac{\mathrm{dD}}{d\tau }=k_{2}L(D+2N-T+2[A_{2}])\\ \frac{d\Phi_{\mathrm{ss}}}{d\tau }=-(D+2N-T+2[A_{2}])(2k_{2}\Phi_{\mathrm{ss}}-4k_{1}\Phi_{\mathrm{ps}})+(\Phi_{\mathrm{ns}}+2L-\Phi_{\mathrm{ps}})(4k_{1}T+4k_{1}\Phi_{\mathrm{ps}}+2k_{2}\Phi_{\mathrm{ss}})\\ \frac{d\Phi_{\mathrm{sp}}}{d\tau }=-(D+2N-T+2[A_{2}])(2k_{1}\Phi_{\mathrm{ps}}+k_{2}\Phi_{\mathrm{ps}}-2k_{1}\Phi_{\mathrm{pp}})+(\Phi_{\mathrm{ns}}+2L-\Phi_{\mathrm{ps}})(2k_{1}\Phi_{\mathrm{pp}}+k_{2}\Phi_{\mathrm{ps}})+\\ +(2k_{1}T+2k_{1}\Phi_{\mathrm{ps}}+k_{2}\Phi_{\mathrm{ss}})(\Phi_{\mathrm{np}}+T-\Phi_{\mathrm{ps}})\\ \frac{d\Phi_{\mathrm{sn}}}{d\tau }=-(D+2N-T+2[A_{2}])(k_{2}\Phi_{\mathrm{sn}}-2k_{1}\Phi_{\mathrm{pn}}-k_{2}\Phi_{\mathrm{ss}})+(\Phi_{\mathrm{ns}}+2L-\Phi_{\mathrm{ps}})(k_{2}L+2k_{1}\Phi_{\mathrm{pn}}+k_{2}\Phi_{\mathrm{ns}})+\\ +(2k_{1}T+2k_{1}\Phi_{\mathrm{ps}}+k_{2}\Phi_{\mathrm{ss}})(3D+\Phi_{\mathrm{nn}}-\Phi_{\mathrm{pn}})\\ \frac{d\Phi_{\mathrm{pp}}}{d\tau }=-4k_{1}\Phi_{\mathrm{pp}}(D+2N-T+2[A_{2}])+(\Phi_{\mathrm{np}}+T-\Phi_{\mathrm{ps}})(4k_{1}\Phi_{\mathrm{pp}}+2k_{2}\Phi_{\mathrm{sp}})\\ \frac{d\Phi_{\mathrm{pn}}}{d\tau }=-(D+2N-T+2[A_{2}])(2k_{1}\Phi_{\mathrm{pn}}-k_{2}\Phi_{\mathrm{sp}})+(\Phi_{\mathrm{np}}+T-\Phi_{\mathrm{ps}})(2k_{1}\Phi_{\mathrm{pn}}+k_{2}L+k_{2}\Phi_{\mathrm{sn}})+\\ +(2k_{1}\Phi_{\mathrm{pp}}+k_{2}\Phi_{\mathrm{sp}})(3D+\Phi_{\mathrm{nn}}-\Phi_{\mathrm{pn}})\\ \frac{d\Phi_{\mathrm{nn}}}{d\tau }=2k_{2}\Phi_{\mathrm{sn}}(D+2N-T+2[A_{2}])+(3D+\Phi_{\mathrm{nn}}-\Phi_{\mathrm{pn}})(4k_{1}\Phi_{\mathrm{pn}}+2k_{2}\Phi_{\mathrm{sn}}+2k_{2}L)\\ with\;initial\;conditions\;being:\\ \left\{\begin{array}{c} N=[AB_{2}{]}_{0}+[B_{4}{]}_{0}\\ T=[AB_{2}{]}_{0}+2[B_{4}{]}_{0}\\ D+2N-T=[AB_{2}{]}_{0}\\ {}[A_{2}]=[A_{2}{]}_{0}\\ L=0\\ D=0 \end{array}\right.\Rightarrow \left\{\begin{array}{c} \left[A_{2}{]}_{0}=[A_{2}\right]\\ {}[AB_{2}{]}_{0}=2N-T\\ {}[B_{4}{]}_{0}=T-N \end{array}\right. \end{array}\right.$$

If R(*l*,*t*,*d*) is the content of macromolecules of the given composition, then the following set of equations can be defined (9):

(9) $$\begin{array}{c} L_{n}\equiv \frac{\sum_{l,t,d}\mathrm{lR}(l,t,d)}{\left(N+[A_{2}]\right)}=\frac{\Phi_{s}\left(1,1,1\right)}{\left(N+[A_{2}]\right)},\\ T_{n}\equiv \frac{\sum_{l,t,d}\mathrm{tR}(l,t,d)}{\left(N+[A_{2}]\right)}=\frac{\Phi_{p}\left(1,1,1\right)}{\left(N+[A_{2}]\right)},\\ D_{n}\equiv \frac{\sum_{l,t,d}\mathrm{dR}(l,t,d)}{\left(N+[A_{2}]\right)}=\frac{\Phi_{n}\left(1,1,1\right)}{\left(N+[A_{2}]\right)} \end{array}$$

where *L_n_*, *T_n_*, and *D_n_* are equal to the values of the average content of linear, terminal, and dendritic units in a macromolecule.

We can determine the value of the average degree of polymerization (DP_n_) as n + 1 amount of B groups involved in the reaction, i.e., the number of monomer units contained in a macromolecule, which is *2d* + *l* + 1. Thus, DP_n_ can be defined as follows (10):

(10) $$DP_{n}=\frac{\sum_{l,t,d}\left(2d+l+1)R(l,t,d\right)}{\left(N+[A_{2}]\right)}=L_{n}+2D_{n}+1$$

The mass average structural parameters can be determined by (11):

(11) $$\begin{array}{c} L_{w}=\frac{\sum_{l,t,d}l^{2}R(l,t,d)}{\sum_{l,t,d}\mathrm{lR}(l,t,d)}=\frac{\Phi_{\mathrm{ss}}\left(1,1,1\right)}{\Phi_{s}\left(1,1,1\right)}+1,\\ T_{w}=\frac{\sum_{l,t,d}t^{2}R(l,t,d)}{\sum_{l,t,d}\mathrm{tR}(l,t,d)}=\frac{\Phi_{\mathrm{pp}}\left(1,1,1\right)}{\Phi_{p}\left(1,1,1\right)}+1,\\ D_{w}=\frac{\sum_{l,t,d}d^{2}R(l,t,d)}{\sum_{l,t,d}\mathrm{dR}(l,t,d)}=\frac{\Phi_{\mathrm{nn}}\left(1,1,1\right)}{\Phi_{n}\left(1,1,1\right)}+1, \end{array}$$

where *L_w_*, *T_w_*, and *D_w_* are the weight average compositions of linear, terminal, and dendritic units in a macromolecule.

The weighted average degree of polymerization (DP_w_) can therefore be estimated by Equation (12):

(12) $$DP_{w}=\frac{\sum_{l,t,d}(2d+l+1{)}^{2}R(l,t,d)}{\sum_{l,t,d}\left(2d+l+1)R(l,t,d\right)}=\frac{4\Phi_{\mathrm{nn}}(1,1,1)+4\Phi_{\mathrm{ns}}(1,1,1)+\Phi_{\mathrm{ss}}(1,1,1)+6\Phi_{d}(1,1,1)+2\Phi_{s}\left(1,1,1\right)}{2\Phi_{n}(1,1,1)+\Phi_{s}(1,1,1)+N}+1$$

The condition of DP_w_ → ∞, which is equivalent to PDI → ∞ (where PDI is a polydispersity index), can be considered a gelation criterion. The degree of branching is defined as the ratio of an actual number of branched units to the maximum possible number of these units in a macromolecule. Here, the branched units are dendritic, so DB can be determined by the following Equation (13) [16]:

(13) $$\mathrm{DB}=\frac{2D}{2D+L}$$

To conclude, the application of the structural kinetic model of AB_2_+A_2_+B_4_ monomer mixture polyaddition enables the study of how p_g_ and various structural and molecular weight characteristics are influenced by each reaction component, as well as by substitution effects, which were impossible to analyze in previous studies.

Nevertheless, at first, it is essential to provide the verification of the investigated model.

### 2.2. Verification of the Kinetic—Structural Model

The current model for AB_2_+A_2_+B_4_ monomer mixture polyaddition is quite versatile, encompassing all systems based on various combinations of the studied monomers, namely, A_2_+AB_2_, AB_2_+B_4_, AB_2_, and A_2_+B_4_. This significantly expands the range of applications for the developed approach, enabling the use of well-known systems and solitary cases, such as A_2_+B_4_, AB_2_, and A_2_+CB_2_, for verification.

**Figure 2:**
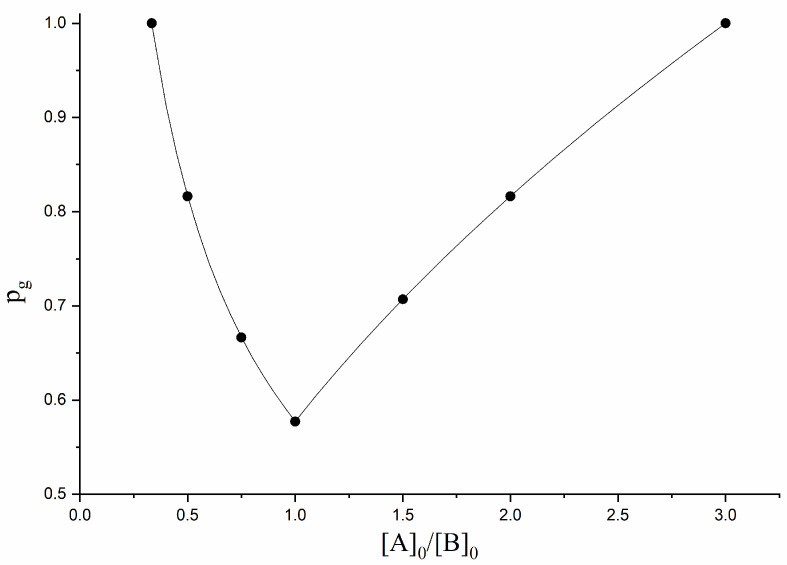
Plot of p_g_ as a function of [A]_0_/[B]_0_ for A_2_+B_4_ system. Solid line depicts the data obtained through Equation (1), and dots represent the data calculated by the offered approach ([AB_2_]_0_ = 0).

The A_2_+B_4_ system is a subset of the A_n_+B_m_ system, which was studied and described by Flory, resulting in Equation (1) [35]. Comparison of the data obtained through (1) and the data calculated using the offered approach (initial conditions are N = [B_4_]_0_, [A_2_] = [A_2_]_0_, T = 2[B_4_]_0_, Φ_pp_ = 2[B_4_]_0_, others are equal to 0) is shown in Figure 2.

As we can clearly see from Figure 2, there is a perfect correlation between data obtained through two different methods.

**Figure 3:**
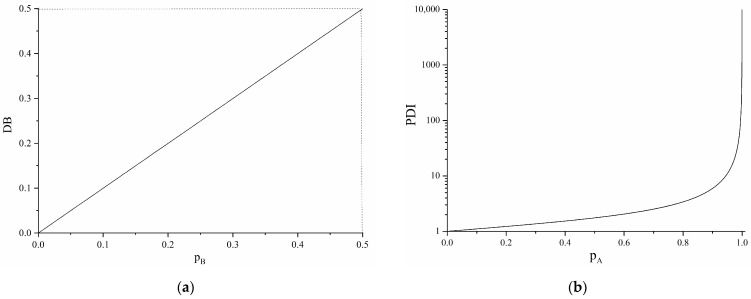
Plot of DB as a function of p_B_ (**a**); plot of PDI as a function of p_A_ (**b**) in the AB_2_ monomer-based system ([AB_2_]_0_ = 1, [A_2_]_0_ = [B_4_]_0_ = 0).

Another method of verification lies in reviewing well-studied systems—one of them is a solitary AB_2_-type monomer. The variations in system characteristics calculated using our method (with initial conditions set as N = [AB_2_]_0_, T = [AB_2_]_0_, and the rest as zero) are illustrated in Figure 3.

Figure 3 shows that the maximum value of DB is 0.5 at p_B_ = 0.5, which corresponds to data from earlier papers [16]. Along with that, the gel point (PDI → ∞ or DP_w_ → ∞) is achieved at p_A_ → 1 (p_B_ → 0.5), which is the same as in a conventional Flory paper [15].

**Figure 4:**
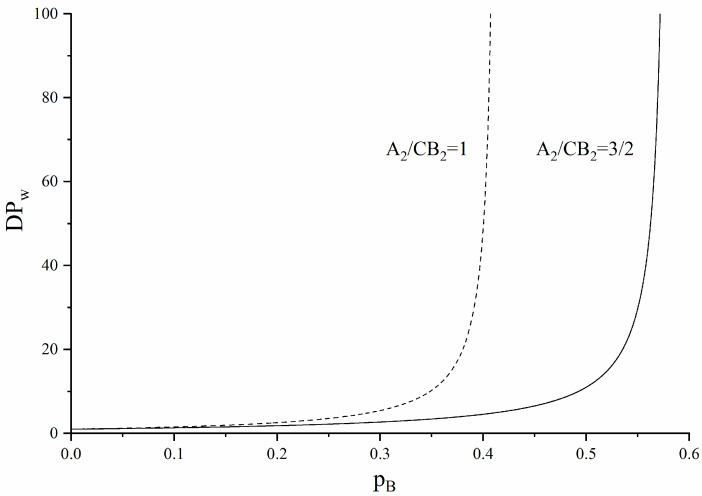
Plot of DP_w_ as a function of p_B_, where the dashed line represents the [AB_2_]_0_/[A_2_]_0_/[B_4_]_0_ = 2/1/1 ([A_2_]_0_/[CB_2_]_0_ = 1) case, and [AB_2_]_0_/[A_2_]_0_/[B_4_]_0_ = 4/4/1 ([A_2_]_0_/[CB_2_]_0_ = 3/2) is for the solid line.

The validation of the comprehensive AB_2_+A_2_+B_4_ model, incorporating all constituents, involves comparing the results obtained with our model to those obtained from the following set of reactions: A_2_+CB_2_→AB_2_ (rate constant k_c_) and AB_2_+CB_2_→B_4_ (rate constant k_b_). For example, from [41], when k_c_/k_b_ = 200, the p_g_ value equals 0.40 for the [A_2_]_0_/[CB_2_]_0_ = 1 ratio and 0.56 for the [A_2_]_0_/[CB_2_]_0_ = 3/2 ratio, respectively. If [A_2_]_0_/[CB_2_]_0_ = 1, the mixture of [AB_2_]_0_/[A_2_]_0_/[B_4_]_0_ at a ratio of 2/1/1 is produced, whereas it is 4/4/1 for the [A_2_]_0_/[CB_2_]_0_ =3/2 case. The DP_w_ values for these mixture compositions, obtained with our suggested approach, are shown in Figure 4 and are similar to the ones specified in [41].

**Figure 5:**
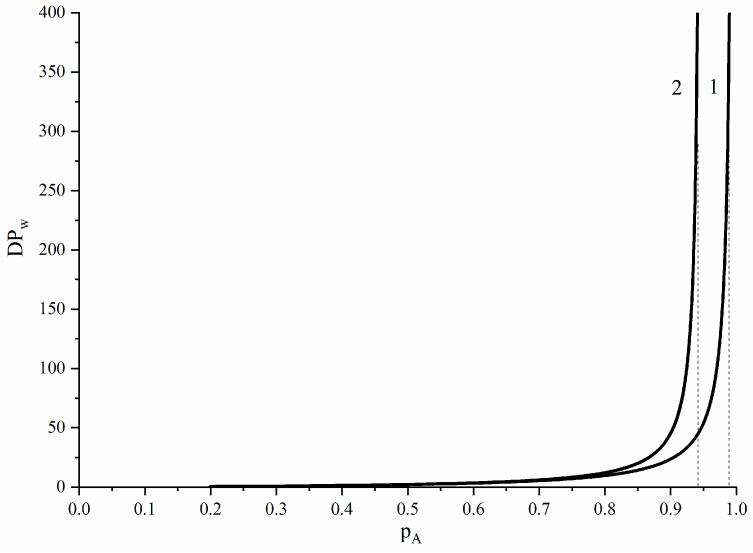
Plot of DP_w_ vs. p_A_ when (1) [AB_2_]_0_/[A_2_]_0_/[B_4_]_0_ = 1/0.025/0.097, or (2) [AB_2_]_0_/[A_2_]_0_/[B_4_]_0_ = 1/0.036/0.083. Dashed lines correspond to the p_A_ values of 0.94 and 0.99.

Experimental data confirm that the offered model describes the polyaddition of AB_2_+A_2_+B_4_ monomers properly. In [43], AB_2_+A_2_+B_4_ monomer mixtures of various compositions were synthesized, and it was determined experimentally that p_g_ value accounts for less than 1 in the range of [AB_2_]_0_/[A_2_]_0_/[B_4_]_0_ ratios from 1/0.025/0.097 to 1/0.036/0.083. Figure 5 illustrates that the first case is characterized by a calculated p_g_ value of ~0.99, while for the second one, the calculated value equals p_g_~0.94.

Thus, the data obtained from various sources and the results of calculation using our suggestions matched perfectly. Based on that, it can be concluded that our structural kinetic model of the polyaddition of AB_2_+A_2_+B_4_ monomer mixture provides accurate results.

## 3. Results and Discussion

Using the proposed approach, it is possible to evaluate the effect of each constituent on both the structure and molecular weight parameters.

### 3.1. A_2_-Type Monomer Effects

**Figure 6:**
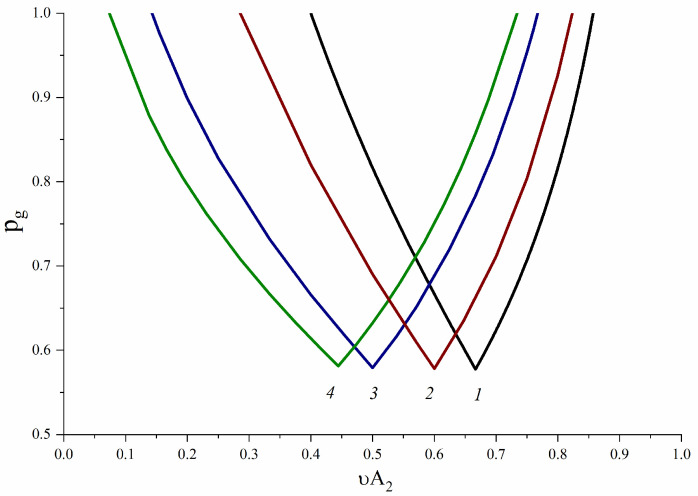
Plot of p_g_ as a function of υA_2_, where (1) [AB_2_]_0_/[B_4_]_0_ = 0 (a curve derived from Flory equation); (2) [AB_2_]_0_/[B_4_]_0_ = 0.5; (3) [AB_2_]_0_/[B_4_]_0_ = 2; and (4) [AB_2_]_0_/[B_4_]_0_ = 4.

The p_g_ curves over the initial molar fraction of an A_2_-type monomer (υA_2_ = [A_2_]_0_/([AB_2_]_0_ + [A_2_]_0_ + [B_4_]_0_)) at different [AB_2_]_0_/[B_4_]_0_ ratios are shown in Figure 6.

**Figure 7:**
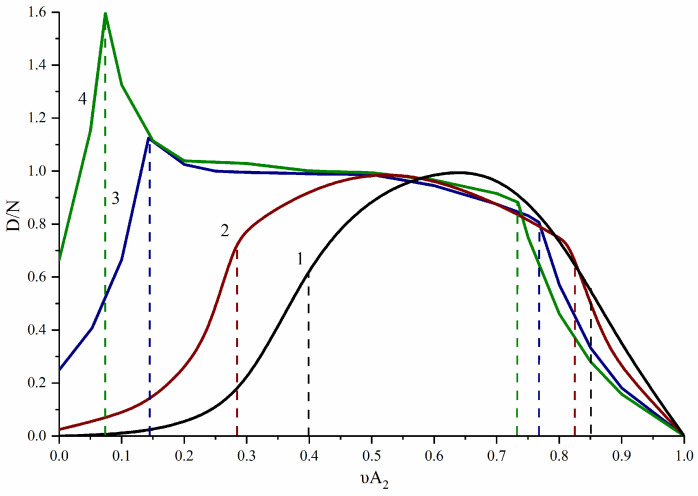
Plot of specific number of branches per macromolecule (D/N) at p_g_ vs. υA_2_ when (1) [AB_2_]_0_/[B_4_]_0_ = 0 (Flory curve); (2) [AB_2_]_0_/[B_4_]_0_ = 0.5; (3) [AB_2_]_0_/[B_4_]_0_ = 2; and (4) [AB_2_]_0_/[B_4_]_0_ = 4. Dashed lines correspond to the points where p_g_ ≤ 1.

The curves in Figure 6 reflect the conditions under which one can observe soluble systems transition to an insoluble state. Here, the condition for curve 1 is [AB_2_]_0_ = 0, indicating that it can be described by Equation (1). In other cases, [AB_2_]_0_ ≠ 0 (Figure 6 (2–4)), and therefore, a broadening of the Flory curve can be observed. Also, there is a distinct minimum at the [A]_0_/[B]_0_ = 1 ratio in all the p_g_ vs. υA_2_ graphs. When the [A]_0_/[B]_0_ value tends to deviate from 1 in either direction, an increase in p_g_ up to 1 is observed. The minimum point shifts towards lower υA_2_ values when an AB_2_-type monomer is introduced into the system. At the same time, the p_g_ value at the minimum point is almost unaffected by changes in the [AB_2_]_0_/[B_4_]_0_ ratio and remains approximately (1/3)^1/2^. To understand the reasons for these observed patterns, it is necessary to analyze how υA_2_ affects the specific number of branches per macromolecule (D/N) (Figure 7). Hereinafter, the values of p_g_ at the corresponding values of υA_2_ were used to calculate the D/N.

It can be observed in Figure 7 (1) that for the polyaddition of A_2_+B_4_ monomers, the specific number of branches per macromolecule increases with the growth of υA_2_ until it reaches 1, corresponding to a minimum of the p_g_ vs. υA_2_ function (Figure 6). As expected, it then begins to decrease. Thus, the minimum p_g_ value is reached when D/N = 1.

**Figure 8:**
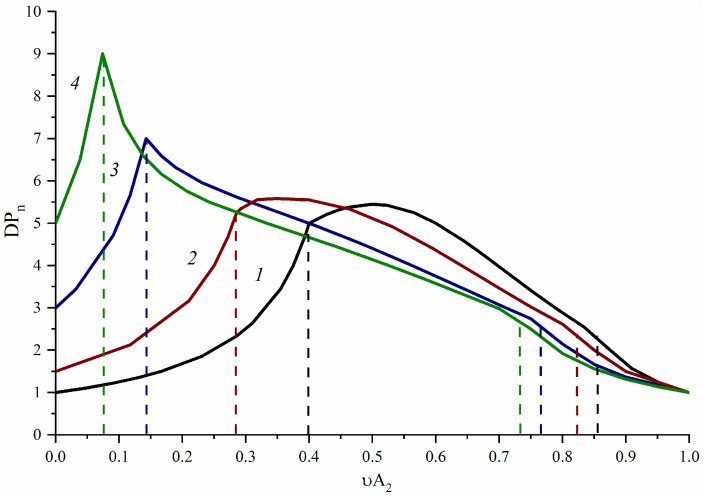
Plot of DP_n_ at p_g_ as a function of υA_2_: (1) [AB_2_]_0_/[B_4_]_0_ = 0 (Flory curve); (2) [AB_2_]_0_/[B_4_]_0_ = 0.5; (3) [AB_2_]_0_/[B_4_]_0_ = 2; and (4) [AB_2_]_0_/[B_4_]_0_ = 4. Dashed lines correspond to the points where p_g_ ≤ 1.

The Introduction of the AB_2_-type monomer into the system leads to an increase in the D/N growth rate over υA_2_. The maximum D/N value possible is 1 when [AB_2_]_0_/[B_4_]_0_ < 1 (Figure 7 (2)), whereas it exceeds 1 at [AB_2_]_0_/[B_4_]_0_ > 1 (Figure 7 (3,4)). Furthermore, the function reaches its maximum when p_g_ ≤ 1. However, the introduction of the AB_2_-type monomer does not affect the condition under which p_g_ reaches its minimum at ~(1/3)^1/2^, which is observed at D/N = 1. Thus, introducing the AB_2_-type monomer into the A_2_+B_4_ system results in an increase in the D/N of the homo-polyaddition of the AB_2_-type monomer and its interaction with the B_4_-type monomer. The mentioned process does not lead to the crosslinking of macromolecules and contributes only to an increase in the degree of polymerization, as indicated by the DP_n_ vs. υA_2_ plots shown in Figure 8.

In the case of polyaddition, the molecular weight of the product depends heavily on the ratio of the groups that are involved in the reaction, and also, the highest molecular weight polymer can only be obtained under equimolar conditions. Another factor affecting the molecular weight is the conversion of functional groups. The effect of conversion on the MW is often complex in nature. In any case, it is obvious that the degree of reaction completion is essential to obtaining a high-molecular-weight polymer.

Where the polyaddition of a binary mixture of A_2_+B_4_ monomers is concerned, there is a correlation between achieving equimolar conditions, a functional group conversion, and the molecular weight of the final product. Due to this, a broad peak is present on the graph of the degree of polymerization as a function of υA_2_ (Figure 8 (1)). The introduction of an AB_2_-type monomer into the system results in shifting the peak (Figure 8 (2)) towards the [A]_0_/[B]_0_ < 1 area. A further increase in this part of the AB_2_-type monomer in the system causes the highest MW to be achieved only when the conversion approaches 1, thereby sharpening the peak (Figure 8 (3,4)). Thus, the increase in υAB_2_ in the AB_2_+A_2_+B_4_ system significantly enhanced the DP_n_ of the final polymer from 5 to 9, with [AB_2_]_0_/[B_4_] changing from 0 to 4; also, υAB_2_ → 1, and DP_n_ → ∞.

**Figure 9:**
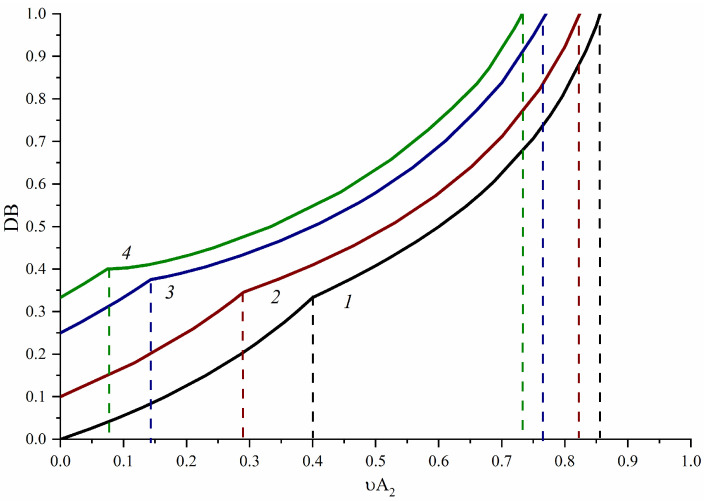
Plot of DB at p_g_ as a function of υA_2_: (1) [AB_2_]_0_/[B_4_]_0_ = 0; (2) [AB_2_]_0_/[B_4_]_0_ = 0.5; (3) [AB_2_]_0_/[B_4_]_0_ = 2; and (4) [AB_2_]_0_/[B_4_]_0_ = 4. Dashed lines correspond to the points where p_g_ ≤ 1.

As expected, a monotonic increase in DB is observed in the curves illustrating its variation over υA_2_, as depicted in Figure 9, up to p_g_ ≤ 1. The inflection point indicates the gelation onset. Figure 9 shows that the introduction of AB_2_-type monomer facilitates the DB growth.

Generally, hyperbranched polymers exhibit a DB ≥ 0.4. This value can be reached with all the ratios used within this work. However, when [AB_2_]_0_/[B_4_]_0_ < 4 (Figure 9 (1–3)), the DB value reaches 0.4 beyond the inflection point, that is, when p_g_ < 1 (and when DP_n_ reaches its highest values). On the other hand, at [AB_2_]_0_/[B_4_]_0_ ≥ 4, fully soluble hyperbranched polymers with DB = 0.4 can be obtained (Figure 9 (4)). The highest DB that is possible for the polyaddition of an AB_2_-type monomer is 0.5. However, HBPs with DB > 0.5 can be obtained using a mixture of AB_2_+A_2_+B_4_ monomers. The point is that the application of the monomer mixtures that can potentially help reach DB ≥ 0.4 results in a decrease in the molecular weight characteristics of the final product compared to the polyaddition of an AB_2_-type monomer.

### 3.2. B_4_-Type Monomer Effects

**Figure 10:**
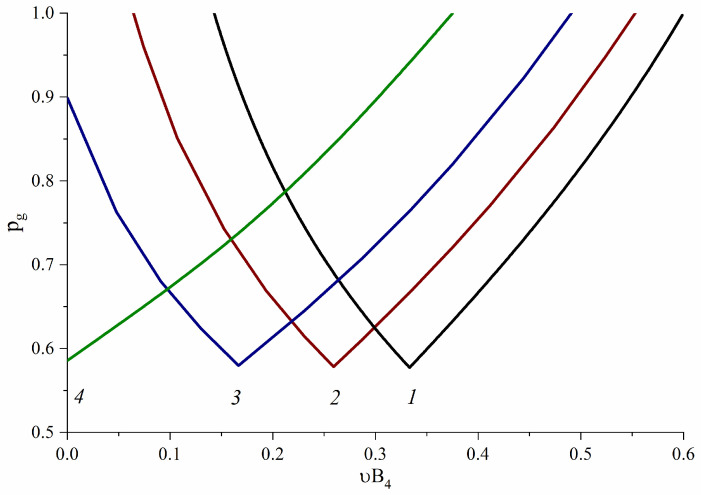
Graph of p_g_ as a function of υB_4_, when (1) [AB_2_]_0_/[A_2_]_0_ = 0 (Flory curve); (2) [AB_2_]_0_/[A_2_]_0_ = 1/4; (3) [AB_2_]_0_/[A_2_]_0_ = 2/3; and (4) [AB_2_]_0_/[A_2_]_0_ = 2.

The next important stage involves investigating how a B_4_-type monomer affects the formation of hyperbranched polymers during the polyaddition of the AB_2_+A_2_+B_4_ monomer mixture. Figure 10 shows that, as in the previous case, the curves of p_g_ over the initial molar fraction of a B_4_-type monomer (υB_4_ = [B_4_]_0_/([AB_2_]_0_ + [A_2_]_0_ + [B_4_]_0_)) tend to broaden when the AB_2_-type monomer is introduced into the system. Also, a distinctive minimum is observed on each curve at [A]_0_/[B]_0_ = 1 for all [AB_2_]_0_/[A_2_]_0_ ratios (Figure 10).

When the polyaddition of the AB_2_+A_2_+B_4_ monomer mixture takes place, a B_4_-type monomer can be introduced into a macromolecule as a linear (when two B-groups in the monomer have reacted) or tri- (when three B-groups in the monomer have reacted) or tetrafunctional (when four B-groups in the monomer have reacted) branching unit. Figure 10 demonstrates that an increase in υB_4_ results in a decrease in p_g_ when [A]_0_/[B]_0_ > 1. This can be explained by an excess of A groups in the system within this range. Here, a B_4_-type monomer is introduced to a macromolecule mainly as a polyfunctional branching unit.

Same as the A_2_-type monomer does, it leads to an increase in the number of branches per macromolecule. A further increase in υB_4_ causes a decrease in both the absolute and specific number of branches per macromolecule, which is associated with the growth of a free B group amount. As a result, the possibility of forming a three-dimensional grid is significantly diminished. The decrease in the number of branches per macromolecule is related to a decrease in the number of reactive A groups. The latter causes an increase in the number of macromolecules, resulting in the trend for short-chain linear polymers to form.

**Figure 11:**
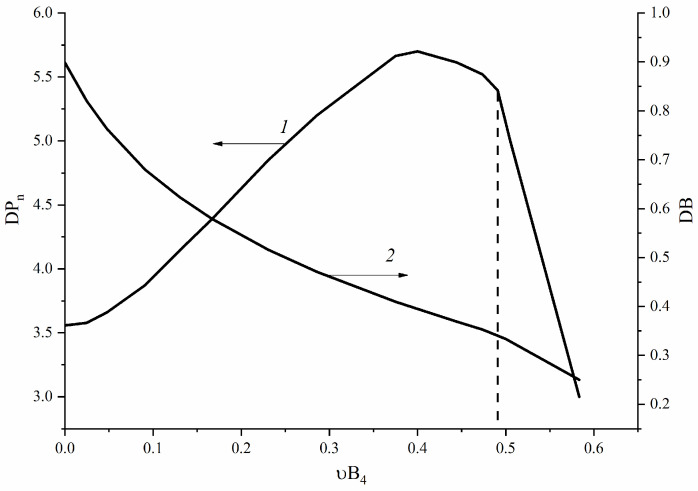
Plot of DP_n_—1 and DB—2 vs. υB_4_, with [AB_2_]_0_/[A_2_]_0_ = 2/3, and conversion is equal to p_g_. Dashed line corresponds to the point where p_g_ ≤ 1.

Thus, subject to [A]_0_/[B]_0_ > 1, a B_4_-type monomer is introduced to a chain mainly as a polyfunctional branching unit; in other words, it acts as a core for a macromolecule to form and grow. Meanwhile, at [A]_0_/[B]_0_ < 1, the monomer is introduced primarily as a linear unit and, eventually, terminates the growing polymer chain (Figure 11).

The graph of DP_n_ and DB vs. υB_4_ is illustrated in Figure 11. As with the A_2_-type monomer, the DP_n_ curve goes through a maximum. However, for the [AB_2_]_0_/[A_2_]_0_ = 2/3 ratio, we can see a broad peak that is related to the area where the gelation is observed. The DB decreases with an increasing υB_4_ due to a decline in the number of cross-linked units. Thus, when no gelation occurs, hyperbranched polymers with B end groups can be obtained, with DB = 0.34 and DP_n_ = 5.4. These characteristic values are not much higher compared to the polyaddition of the A_2_+B_4_ monomer mixture (DB = 0.33 and DP_n_ = 5.0).

### 3.3. AB_2_-Type Monomer Effects

**Figure 12:**
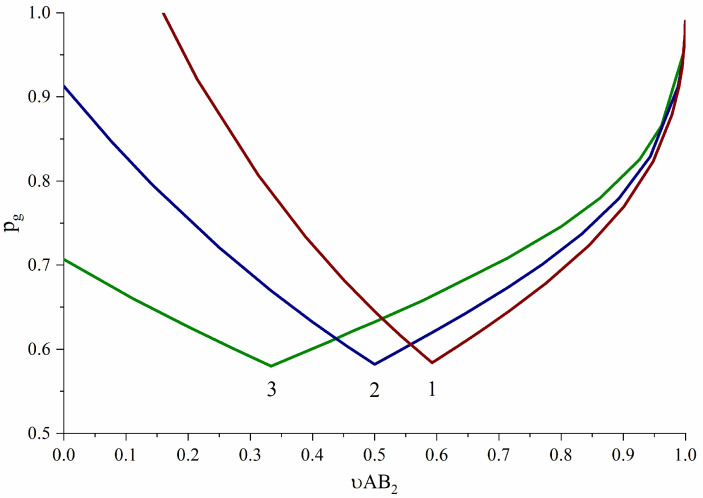
Plot of p_g_ vs. υAB_2_ at (1)—[A_2_]_0_/[B_4_]_0_ = 3; (2) [A_2_]_0_/[B_4_]_0_ = 5; and (3) [A_2_]_0_/[B_4_]_0_ = 10.

The plot of p_g_ over the initial molar fraction of the AB_2_-type monomer (υAB_2_ = [AB_2_]_0_/([AB_2_]_0_ + [A_2_]_0_ + [B_4_]_0_)) is of particular interest (Figure 12). In contrast to the two cases above, there are no distinctive points at which gelation would not be observed, when [A]_0_/[B]_0_ > 1. The p_g_ → 1 only when υAB_2_ → 1, which corresponds to the data from [15]. The minimum of the function is also observed at [A]_0_/[B]_0_ = 1, and shifting from equimolar conditions results in an increase in p_g_. The p_g_ value decreases with an increase in υAB_2_ when [A]_0_/[B]_0_ > 1. The reason lies in the fact that under these conditions, an AB_2_-type monomer can be introduced into the chain mainly as a trifunctional unit, thereby increasing the number of these units per macromolecule and causing a decrease in p_g_. On the other hand, with an excess of B groups ([A]_0_/[B]_0_ < 1), an increase in the AB_2_ monomer content promotes an increase in the number of terminal and linear units in a macromolecule. Thus, an AB_2_-type monomer can be introduced in a growing polymer chain both as a trifunctional and as a linear unit.

**Figure 13:**
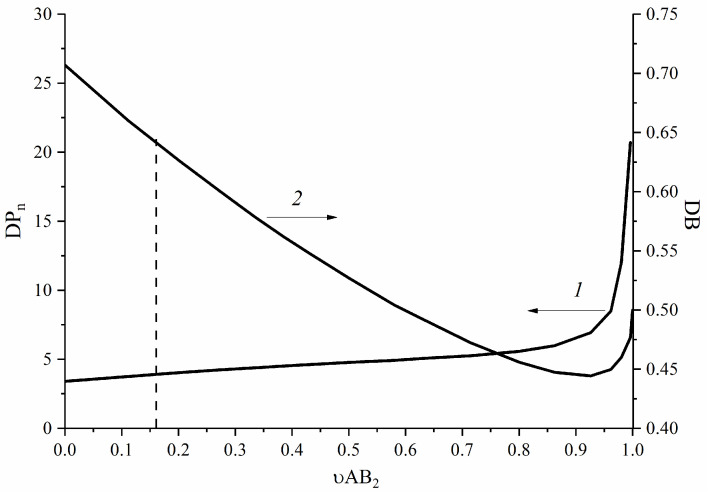
Plot of DP_n_—1 and DB—2 vs. υAB_2_ with [A_2_]_0_/[B_4_]_0_ = 3; conversion is equal p_g_. Dashed line corresponds to the point where p_g_ ≤ 1.

The plots of DP_n_ and DB vs. υAB_2_ are shown in Figure 13. In contrast to all of the aforementioned options, a monotonic increase in DP_n_ is observed with an increase in υAB_2_ over the entire range. Moreover, the curves appear to be almost linear up to υAB_2_ ~ 0.90 due to the contribution of each component of the AB_2_+A_2_+B_4_ monomer mixture to the polyaddition process. Nevertheless, a further increase in υAB_2_ leads to an exponential increase in DP_n_, associated with a negligible contribution of A_2_- and B_4_-type monomers compared to the AB_2_ type. The DB graph reaches its lowest value and then tends to grow at υAB_2_ ~ 0.90 for the exact same reasons.

**Figure 14:**
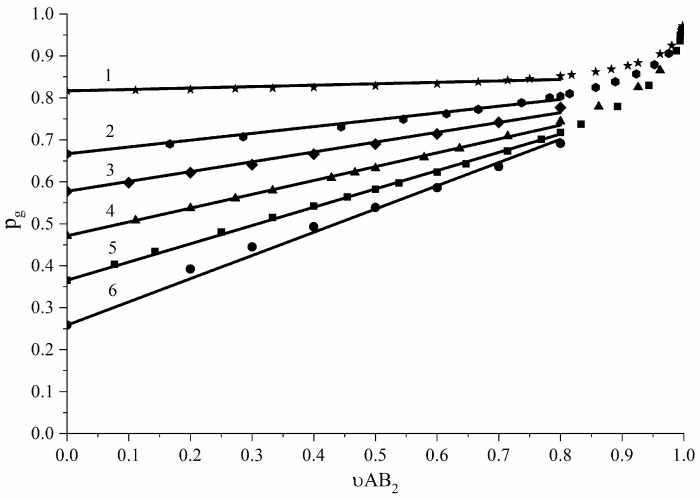
Plot of p_g_ = p_A_ as a function of υAB_2_ at [A_2_]_0_/[B_4_]_0_ ratio equal to (1) 1; (2) 3/2; (3) 2; (4) 3; (5) 5; and (6) 10.

As indicated above, the Flory Equation (1) for p_g_ determination is relevant solely for the polyaddition of A_n_+B_m_ monomers, without taking the AB_2_-type monomer effect into account. To figure out how p_g_ = p_A_ can be influenced by the composition of the AB_2_+A_2_+B_4_ monomer mixture, the curves of p_g_ = p_A_ vs. υAB_2_ were plotted for a range of the [A_2_]_0_/[B_4_]_0_ ratio of 1–10 (Figure 14).

Figure 14 demonstrates that each graph here can be accurately described by the linear equation p_g_ = p_A_ = *a* × υAB_2_ + *b*, where υAB_2_ ranges between 0 and 0.8.

The constant term (*b*) can be determined using the Flory Equation (1) at υAB_2_ = 0. Due to the fact that the parameters *α* and *ρ* are constants for every single case of polyaddition, the correlation between p_g_ = p_A_ and the parameter r = [A]_0_/[B]_0_ will appear as (14). Therefore, the curve of the constant term (*b*) vs. ([B]_0_/[A]_0_)^1/2^ should be linear (see Figure S1a in Supporting Information). Here, [A]_0_ and [B]_0_ represent A and B groups from A_2_ and B_4_, respectively.

(14) $$p_{g}=p_{A}=\sqrt{\frac{\alpha }{r(\rho +\alpha (1-\rho )}}\;\sim \sqrt{\frac{1}{r}}\sim \sqrt{\frac{{\left[B\right]}_{0}}{{\left[A\right]}_{0}}}$$

The slope coefficient (*a*) appears to be influenced by an AB_2_-type monomer introduction; however, the relationship between *a* and ([B]_0_/[A]_0_)^1/2^ also exhibits linearity (see Figure S1b in Supporting Information).

Thus, we can estimate the p_g_ = p_A_ value during the polyaddition of the AB_2_+A_2_+B_4_ monomer mixture through the following Equation (15):

(15) $$p_{g}=p_{A}=(-0.53([B{]}_{0}/[A{]}_{0}{)}^{1/2}+0.78)\upsilon AB_{2}+(1/3{)}^{1/2}([B{]}_{0}/[A{]}_{0}{)}^{1/2}$$

where (1/3)^1/2^ × ([B]_0_/[A]_0_)^1/2^ is Equation (1) for the polyaddition of the A_2_+B_4_ monomer mixture, [A]_0_ and [B]_0_ represent the content of A and B groups from A_2_ and B_4_, respectively. The equation allows for the accurate calculation of the p_g_ = p_A_ value when υAB_2_ is up to 80%.

One of the most significant advantages of the invented model is an opportunity to calculate structural and molecular weight properties while considering substitution effects (Scheme 3).

### 3.4. Substitution Effects

Let us simulate the case of a monomer mixture polyaddition when [AB_2_]_0_/[A_2_]_0_/[B_4_]_0_ = 0.63/0.060/0.31, based on A_2_+B′B_2_ and A_2_+CB_2_ polyaddition cases. The impact of the *k*_2_/*k*_1_ ratio on the structural and molecular weight parameters of the hyperbranched polymers that are obtained under these conditions is illustrated in Figures S2 and S3 (Supporting Information), respectively.

As we can see from Figure S2 (Supporting Information), the negative substitution effect leads to DB → 0. That is, the topological mechanism of the macromolecule formation changes drastically, resulting in the formation of weakly branched polymers with numerous side-chained B groups. It seems nearly impossible to obtain hyperbranched polymers under these conditions. On the contrary, when *k*_2_/*k*_1_ > 1, the possibility of forming knots increases the same way that the ratio does, causing an increase in the DB.

As we expected, DP_n_ is unaffected by the presence of the substitution effect (see Figure S3 in Supporting Information). It is evident that, when no gelation occurs, the *k*_2_/*k*_1_ ratio has no impact on the completion of the process. We can conclude that DP_n_ is indifferent to the unequal reactivity of groups, unlike DP_w_. As the *k*_2_/*k*_1_ ratio grows, an increase in the possibility of generating dendritic units can be observed. Thus, there is a higher chance of obtaining high-molecular-weight macromolecules, causing DP_w_ to increase.

The derived regularities are expected for any values of the [A_2_]_0_/[AB_2_]_0_/[B_4_]_0_ ratio. However, each component of the system has a different impact on the forming of hyperbranched polymers.

**Figure 15:**
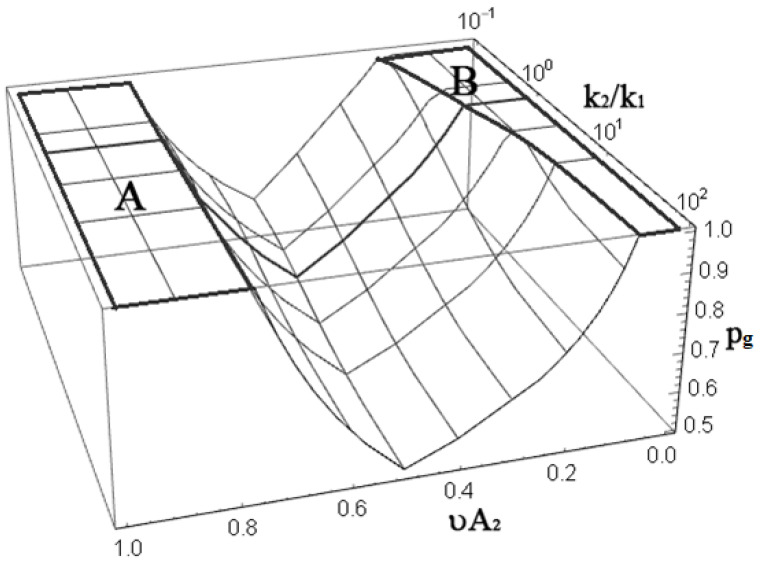
Plot of p_g_ vs. υA_2_ and *k*_2_/*k*_1_ if [AB_2_]_0_/[B_4_]_0_ = 2.

A joint influence of the substitution effect and υA_2_ on p_g_, when [AB_2_]_0_/[B_4_]_0_ = 2, is shown in Figure 15. It illustrates that the positive substitution effect, i.e., when *k*_2_/*k*_1_ > 1, leads to an decrease in p_g_ compared to the statistical polyaddition of an AB_2_+A_2_+B_4_ monomer mixture. For instance, if υA_2_ = 0.14 and *k*_2_/*k*_1_ = 1, p_g_ takes a value of 1, whereas it reaches 0.86 when *k*_2_/*k*_1_ = 10. Both positive and negative substitution effects modify the topological mechanism of macromolecule formation. At the initial stage of the polyaddition of an AB_2_+A_2_+B_4_ monomer mixture, when *k*_2_/*k*_1_ > 1, macromolecules with numerous dendritic units are mainly formed. These macromolecules are characterized by an enhanced content of B groups, which act as cross-linking centers, causing them to form a three-dimensional mesh. In contrast, when the negative substitution effect (*k*_2_/*k*_1_ < 1) takes place, the formation of polymers with numerous linear units is primarily observed during the entire process. This is attributed to the lower reactivity of B groups within linear fragments that is characteristic of this specific case. Therefore, the cross-linked polymer is less likely to form compared to the statistical polyaddition of the AB_2_+A_2_+B_4_ monomer mixture.

When p_g_ reaches 1, an inflection appears in the surface of the graph due to the cessation of changes in p_g_. Thus, we can define an area in the graph that is depicted in Figure 15, which is limited by inflection points where υA_2_ and *k*_2_/*k*_1_ can be adjusted freely, named the ‘sustainability area’. There are no restrictions imposed on the polyaddition of the AB_2_+A_2_+B_4_ monomer mixture and associated with the gelation process in the so-called ‘sustainability area’, since the formation of a three-dimensional spatial network here is impossible. With υA_2_ values being high, fully branched polymers with terminal A groups and relatively low DP_n_ values can be produced, as indicated by the relations derived above. On the other hand, for lower υA_2_ values, we can obtain hyperbranched polymers with terminal B groups, exhibiting a DB close to or exceeding 0.5 and a relatively high molecular weight. Similar plots can be derived for each monomer in the AB_2_+A_2_+B_4_ ternary system. Thus, by varying the component composition and/or *k*_2_/*k*_1_, it is possible to define a range of the system parameter values where soluble products with predefined structural and molecular weight parameters are formed consistently.

## 4. Conclusions

A new structural kinetic model of the polyaddition of an AB_2_+A_2_+B_4_ monomer mixture was designed within this work in order to predict the impact of the composition of the monomer mixture on the structural (DB) and molecular weight (DP_n_, DP_w_) characteristics of HBPs, as well as p_g_. The suggested model also considers a positive or negative substitution effect to occur during the polyaddition. The relevance of the polyaddition description for the AB_2_+A_2_+B_4_ system was verified by the interaction of well-defined systems, like A_2_+B_4_, AB_2_, and A_2_+CB_2_. Furthermore, p_g_ values obtained using the proposed model are in agreement with the experimental data that are derived from the scientific sources that are dedicated to the polyaddition of AB_2_+A_2_+B_4_ monomers.

Using the suggested model, the influence of both the component’s composition and the substitution effect during the polyaddition of AB_2_+A_2_+B_4_ monomers on the structural and molecular weight characteristics of hyperbranched polymers was investigated.

It was shown that with an increase in the A_2_-type monomer content in the ternary system under study, the value of p_g_ decreases. This is also accompanied by an increase in DP_n_ and DB as a result of the cross-linking of macromolecules formed at the initial stages, containing B groups in terminal and linear units. The introduction of a B_4_-type monomer into the AB_2_+A_2_ monomer system also leads to a decrease in the p_g_ value, accompanied by an increase in DP_n_ and a decrease in DB, as a result of the cross-linking of macromolecules that are also formed at the initial stages, containing terminal A groups. In both cases, the maximum values of DP_n_ and DB belong to the area where [A]_0_/[B]_0_ < 1. It should be noted that the effect of the monomers of the A_2_ and B_4_ type on DP_n_ is extreme. However, when an AB_2_-type monomer is introduced into the A_2_+B_4_ system, the DP_n_ value increases over the entire concentration scale. On the contrary, the DB value decreases to a certain limit when the AB_2_ monomer concentration approaches 90%, after which it begins to increase. Thus, when the amount of AB_2_-type monomers is less than 90%, the contribution of each constituent of the AB_2_+A_2_+B_4_ system to polyaddition is comparable. In contrast, with an AB_2_ monomer content exceeding 90%, the contribution of the A_2_ and B_4_ types of monomers becomes negligible.

Based on our results, an empirical formula has been proposed for estimating p_g_ for the polyaddition of an AB_2_+A_2_+B_4_ monomer mixture: p_g_ = p_A_ (−0.53([B]_0_/[A]_0_)^1/2^ + 0.78)υAB_2_ + (1/3)^1/2^([B]_0_/[A]_0_)^1/2^, where (1/3)^1/2^([B]_0_/[A]_0_)^1/2^ represents a Flory equation for the case of A_2_+B_4_ polyaddition; [A]_0_ and [B]_0_ are concentrations of groups A and B from A_2_ and B_4_, respectively; and υAB_2_ represents the mole fraction of AB_2_-type monomers in the mixture. The resulting equation is able to predict precisely the p_g_ value at AB_2_ monomer contents up to 80%.

The presence of ‘sustainability areas’ is shown, where it is possible to freely vary all the system variables and to obtain soluble hyperbranched polymers with various sets of the functional end groups.

It is revealed that the range of initial monomer ratios, where soluble products of the highest molecular weight possible can be obtained, increases in case of a negative substitution effect. Moreover, linear polymers with side-chained B groups tend to form. As the negative effect of substitution increases, the degree of branching approaches 0. In contrast, the positive substitution effect is accompanied by the trend to form polymers with more functional groups in terminal units, with the degree of branching approaching 1. In this case, however, a narrowing of the ‘sustainability area’ occurs.

## Data Availability

Data are contained within the article.

## Supplementary Materials

The following supporting information can be downloaded at https://www.mdpi.com/article/10.3390/polym16030426/s1: Figure S1: Plot of the constant term *b* (**a**) and the slope coefficient *a* (**b**) as a function of ([B_0_]/[A_0_])^1/2^. Figure S2. Plot of DB vs. *k*_2_/*k*_1_; the ratio of monomer mixture polyaddition [AB_2_]_0_/[A_2_]_0_/[B_4_]_0_ = 0.63/0.060/0.31. Figure S3. Plot of DP_n_—1 and DP_w_—2 vs. *k*_2_/*k*_1_ ratio during the polyaddition of the monomer mixture [AB_2_]_0_/[A_2_]_0_/[B_4_]_0_ = 0.63/0.060/0.31.
