# Supplementary material for: A Theoretical Investigation of the Polyaddition of an AB2+A2+B4 Monomer Mixture
Source: Polymers (Basel). 2024 Feb 3;16(3):426. doi: 10.3390/polym16030426 (PMC10857212; doi:10.3390/polym16030426)
Supplement: Supplementary file 1 [file polymers-16-00426-s001.zip › polymers-2785601-supplementary.pdf]

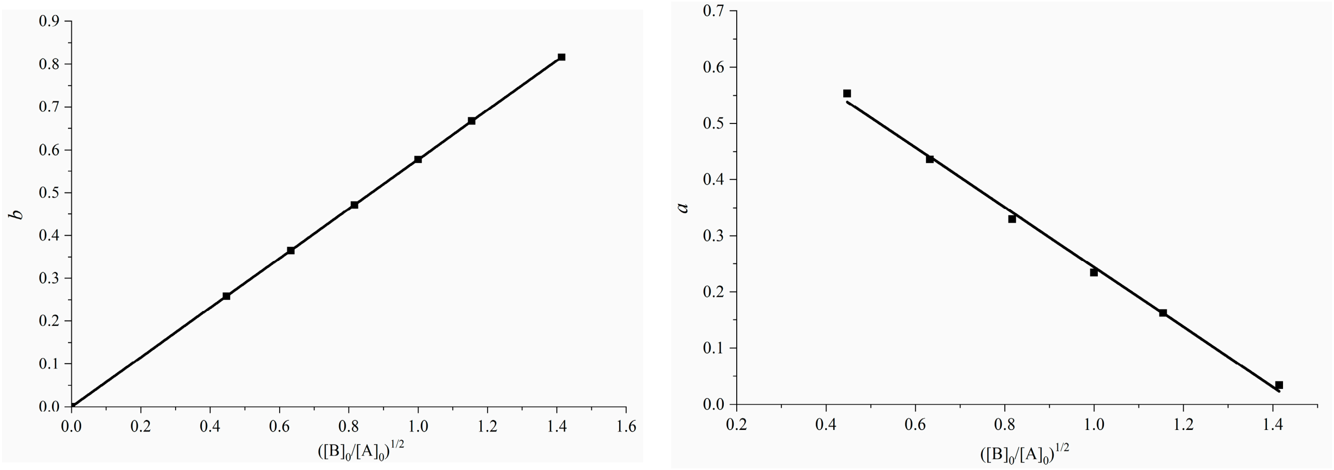

(**a**) (**b**)

Figure S1. Plot of the constant term *b* **(a)** and the slope coefficient *a* **(b)** as a function of $([B_0]/[A_0])^{1/2}$.

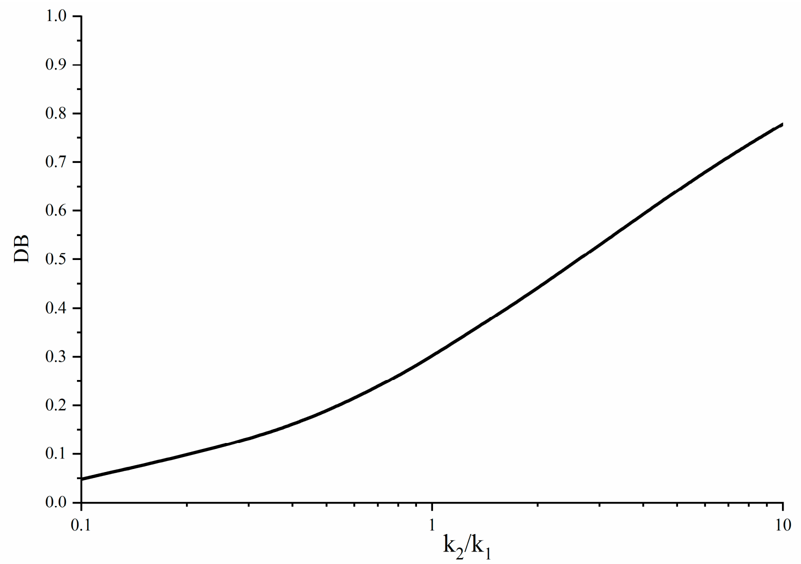

Figure S2. Plot of DB vs k_2/k_1; the ratio of monomer mixture polyaddition $[AB_2]_0/[A_2]_0/[B_4]_0$ = 0.63/0.060/0.31.

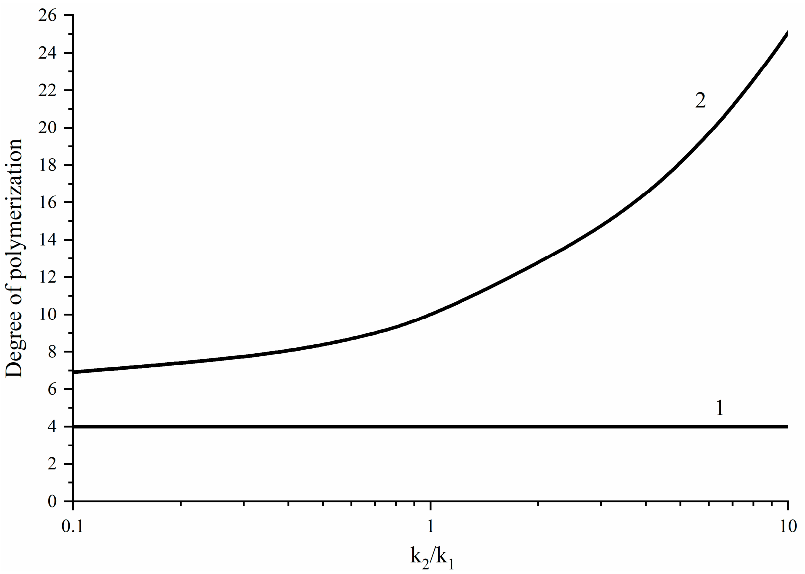

Figure S3. Plot of DP_n – *1* and DP_w – *2* vs k_2/k_1 ratio during the polyaddition of the monomer mixture $[AB_2]_0/[A_2]_0/[B_4]_0$ = 0.63/0.060/0.31.
